# Passive immunization with anti-ActA and anti-listeriolysin O antibodies protects against *Listeria monocytogenes* infection in mice

**DOI:** 10.1038/srep39628

**Published:** 2016-12-22

**Authors:** Krisana Asano, Hiroshi Sashinami, Arihiro Osanai, Shouhei Hirose, Hisaya K. Ono, Kouji Narita, Dong-Liang Hu, Akio Nakane

**Affiliations:** 1 Department of Microbiology and Immunology, Hirosaki University Graduate School of Medicine, 5 Zaifu-cho, Hirosaki, Aomori, 036-8562, Japan; 2 Laboratory of Zoonoses, Kitasato University School of Veterinary Medicine, 35-1 Higashi 23-bancho, Towada, Aomori, 034-8628, Japan; 3 Institute for Animal Experimentation, Hirosaki University Graduate School of Medicine, 5 Zaifu-cho, Hirosaki, Aomori, 036-8562, Japan

## Abstract

*Listeria monocytogenes* is an intracellular pathogen that causes listeriosis. Due to its intracellular niche, *L. monocytogenes* has evolved to limit immune recognition and response to infection. Antibodies that are slightly induced by listerial infection are completely unable to protect re-infection of *L. monocytogenes*. Thus, a role of antibody on the protective effect against *L. monocytogenes* infection has been neglected for a long time. In the present study, we reported that passive immunization with an excessive amount of antibodies against ActA and listeriolysin O (LLO) attenuates severity of *L. monocytogenes* infection. Combination of these antibodies improved survival of *L. monocytogenes* infected mice. Bacterial load in spleen and liver of listerial infected mice and infected RAW264.7 cells were significantly reduced by administration of anti-ActA and anti-LLO antibodies. In addition, anti-LLO antibody neutralized LLO activity and inhibited the bacterial escape from the lysosomal compartments. Moreover, anti-ActA antibody neutralized ActA activity and suppressed actin tail formation and cell-to-cell spread. Thus, our studies reveal that passive immunization with the excessive amount of anti-ActA and -LLO antibodies has potential to provide the protective effect against listerial infection.

*Listeria monocytogenes*, a Gram-positive intracellular pathogen, is environmentally widespread and causes severe food-borne infections in humans and animals^1^. Listeriosis includes encephalomeningitis in immunocompromised hosts and miscarriage in pregnant women. *L. monocytogenes* is able to invade a wide range of cell types, including macrophages, hepatocytes, enterocytes, epithelial cells and endothelial cells. After entry into host cell, *L. monocytogenes* lyses phagosomal vacuole and is released into the cytoplasm^2^. It then replicates and spreads to adjacent cells by mediating actin assembly^3^. During infection, *L. monocytogenes* produces several virulence factors. Its adhesins include fibronectin-binding protein (FbpA), p60 and Ami^4^,^5^,^6^. Internalization into host cell requires invasive proteins, internalin InlA and InlB^7^,^8^. To escape from phagocytic vacuoles, *L. monocytogenes* produces pore-forming listeriolysin O (LLO)^9^ and phospholipase C (PI-PLC)^10^,^11^. This bacterium also produces ActA, a protein that is required for formation of actin rocket tails as well as for spread of bacteria from cell to cell^12^.

*L. monocytogenes* is an excellent model pathogen to study immune response. At the early stage of infection with *L. monocytogenes*, innate immunity serves an essential role to control bacterial number^13^. Long-term protective immunity to *L. monocytogenes* is entirely mediated by listerial-specific T cells^14^. On the other hand, humoral immunity does not appear to play a significant role in clearance of *L. monocytogenes*^15^. Due to intracellular nature of *L. monocytogenes*, antibody response is slightly induced during primary *L. monocytogenes* infection. Only low levels of antibodies are induced and these antibodies are unable to confer protection during a re-infection with *L. monocytogenes*. Moreover, vaccination with heat-killed *L. monocytogenes* does not provide protective immunity^16^. Therefore, application of protective antibody to *L. monocytogenes* infection is almost omitted. However, antibodies are well known to contribute to immune response against bacterial pathogens. They neutralize their toxins, opsonize bacteria which promote uptake by phagocytic cells, and activate complements which enhance opsonization^17^. Although listerial infection does not generate high titers of antibodies that are protective, a monoclonal antibody against LLO can provide protection by acting intracellularly to neutralize LLO activity^18^. This study suggests that the conventional approach using antibodies to neutralize virulence factors may provide protection against listerial infections.

In this study, specific antibodies against several virulence factors of *L. monocytogenes* were generated from rabbits. The protective effect of these antibodies was observed by passive immunization. Our studies reveal that anti-ActA and anti-LLO antibodies have a significant potential to protect *L. monocytogenes* infection.

## Results

### Passive immunization with anti-ActA and anti-LLO antibodies protects mice from listerial infection

**Figure 1:**
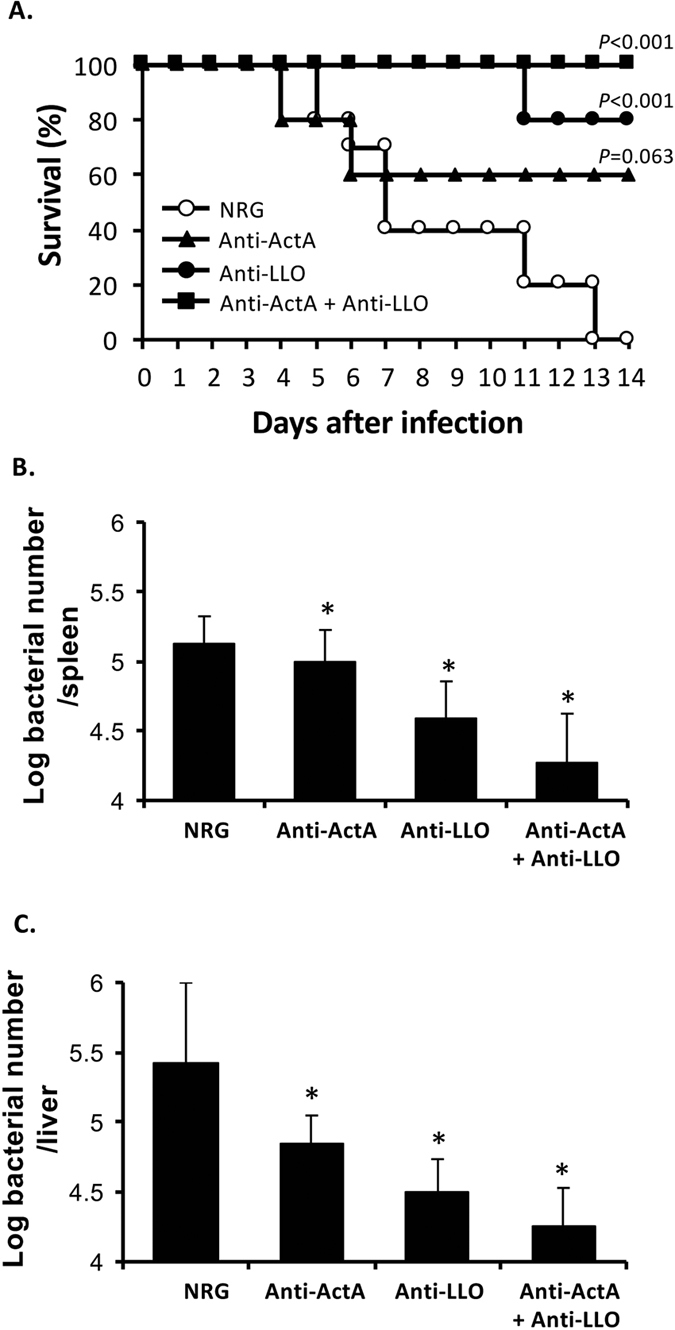
Passive immunization of anti-ActA and anti-LLO antibodies protects mice from listerial infection. Mice were administered intravenously with the antibody or NRG 1 mg/mouse. Mice were infected intravenously with 1 × 10^6^ CFU *L. monocytogenes* 24 h later. (**A**) Survival was observed for 14 days (n = 10). *P*-values calculated by log rank test are indicated. (**B,C**) On day 3 after infection, the bacterial load in the spleens (**B**) and livers (**C**) were enumerated (mean ± S.D. n = 9). *P < 0.05.

Specific antibodies against FbpA, p60, LLO, PI-PLC and ActA were prepared from rabbits. Mice were administered with these antibodies 24 h prior to *L. monocytogenes* infection. Survival of mice was observed for 14 days (see Supplementary Fig. S1A). In comparison to normal rabbit globulin (NRG), survival of listerial infected mice was considerably improved by anti-ActA antibody as well as anti-LLO antibody but not by anti-FbpA, p60 or PI-PLC antibody. These results prompted us to further examine the protective effect of anti-ActA and anti-LLO antibodies. Combination of these antibodies completely improved survival of listerial infected mice (Fig. 1A). This effect remained partially when antibodies were administered after listerial infection for 6 h (see Supplementary Fig. S1B). The results reveal that anti-ActA and anti-LLO antibodies have an impact to protect and treat mice against listerial infection. To determine whether this protective effect requires either interferon-γ (IFN-γ) or tumor necrosis-α (TNF-α)^19^,^20^, experiments using IFN-γ-deficient (IFN-γ^−/−^) and TNF-α-deficient (TNF-α^−/−^) mice were performed (see Supplementary Fig. S2). Although survival of IFN-γ^−/−^ and TNF-α^−/−^ mice was improved by combination of anti-ActA and anti-LLO antibodies, this improvement was considerably reduced in comparison to the wild type mice (Fig. 1A). These results suggest that IFN-γ and TNF-α contribute to the protective effect of anti-ActA and anti-LLO antibodies. The protective effect of anti-ActA and anti-LLO antibodies in the wild type mice was also observed by bacterial load in the organs. On day 3 after infection, bacterial loads in the spleens and livers were significantly reduced by pre-administration with anti-ActA antibody and anti-LLO antibody. Anti-LLO antibody showed more efficient effect than anti-ActA antibody and the most efficient effect was found from the combination of these antibodies (Fig. 1B,C).

### Anti-ActA and anti-LLO antibodies reduce intracellular number of *L. monocytogenes* in murine macrophages

**Figure 2:**
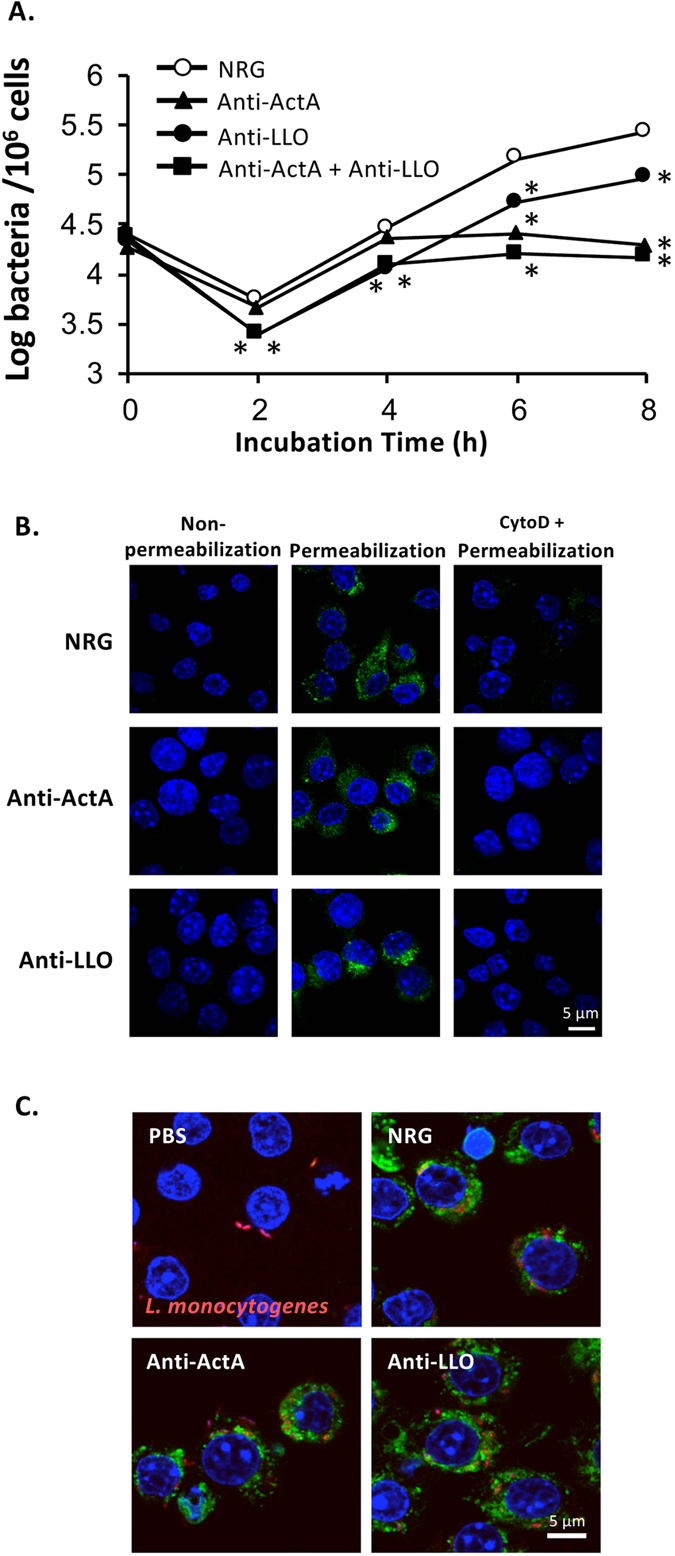
Antibodies internalize into murine macrophages and reduce intracellular number of *L. monocytogenes*. RAW264.7 cells were plated on 24-well culture plates at 1 × 10^6^ cell/well. (**A**) The cells were treated with 100 μg of the antibody or NRG and infected simultaneously with *L. monocytogenes* at MOI 10. After incubation for 30 min, the extracellular bacteria were eliminated with 50 μg/ml gentamicin. At each time point, intracellular bacterial number was enumerated (mean ± S.D., n = 9). *P < 0.01. (**B**) The cells were treated with 100 μg of the antibody or NRG for 2 h. After fixing, immunostaining was performed using AlexaFluor 488-conjugated donkey anti-rabbit IgG. DAPI was used to stain cell nucleus. Left panel: the cells were not permeabilized with Triton X-100, middle and right panels: the cells were permeabilized with Triton X-100, right panel: endocytosis was blocked by cytochalasin D before treatment with the antibody. (**C**) The cells were treated 100 μg of the antibody or NRG or PBS and infected simultaneously with DsRedEx-labeled *L. monocytogenes* at MOI 10. After incubation for 30 min, the extracellular bacteria were washed and eliminated with 50 μg/ml gentamicin. After fixation and permeabilization, immunostaining was performed using AlexaFluor 488-conjugated donkey anti-rabbit IgG. DAPI was used to stain cell nucleus.

We further examined the protective effect of anti-ActA and anti-LLO antibodies *in vitro*. Murine macrophages, RAW264.7 cells, were treated with the antibodies and simultaneously infected with *L. monocytogenes*. Intracellular bacterial number was enumerated at several time points (Fig. 2A). Invasive bacterial number determined at 30 min after gentamicin treatment refers to 0 h of infection. Neither anti-ActA nor anti-LLO antibody significantly modulated bacterial invasion into RAW264.7 cells. However, both antibodies affected intracellular growth of *L. monocytogenes*. Intracellular bacterial number in the cells treated with anti-ActA antibody was significantly reduced from 6 h of infection, whereas bacterial number in the cells treated with anti-LLO antibody was reduced from 2 h. As expected, the most efficient effect was found from the combination of these antibodies, especially at early time points. We confirmed that both antibodies did not significantly modulate bacterial adhesion to RAW264.7 cells (see Supplementary Fig. S3A). Furthermore, involvement of the antibodies to listerial adhesion was examined using murine hepatocytes, NMuLi cells (see Supplementary Fig. S3B). The results demonstrated that these antibodies did not significantly affect bacterial adhesion to murine macrophages and even to non-professional phagocytic hepatocytes.

### The antibodies and NRG efficiently enter into macrophages

Internalization of the antibodies into RAW264.7 cells was examined by immunostaining. Without listerial infection, the antibodies as well as NRG were internalized into macrophages at 2 h after the antibody treatment (Fig. 2B). It should be noted that the detected antibodies (middle panel) were not derived from host cell surface because the signal could not be detected without permeabilization of cell membrane (left panel). Moreover, the antibody internalization was inhibited by cytochalasin D, a drug that induces depolymerization of actin cytoskeleton (right panel). The results suggest that the antibodies and NRG enter into the host cells by endocytosis. Furthermore, when the antibodies or NRG were simultaneously treated to the host cells with *L. monocytogenes*, internalization of the antibodies and NRG was clearly detected in the host cells at 30 min of listerial infection (Fig. 2C). The results demonstrated that the antibodies and NRG efficiently entered into macrophages along with *L. monocytogenes*.

### Neutralizing activity of anti-LLO antibody

**Figure 3:**
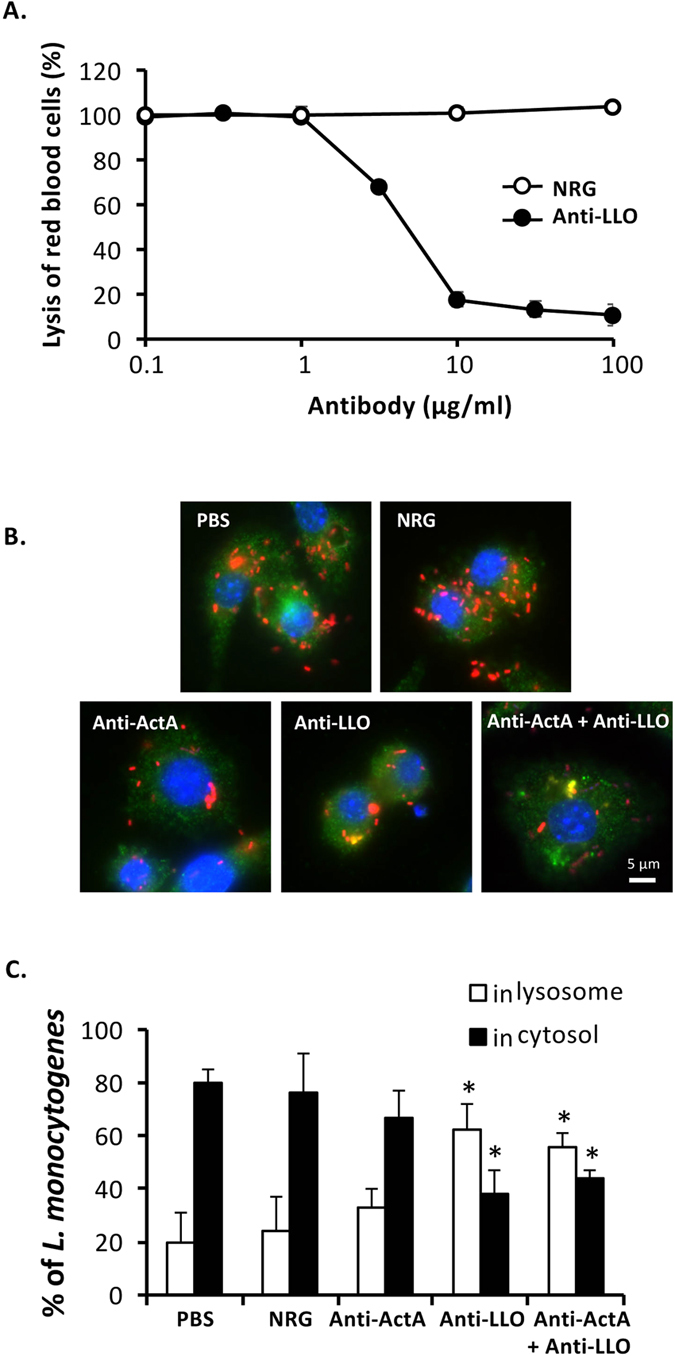
Neutralizing activity of the anti-LLO antibody. (**A**) rLLO (1.0 μg/ml) was pre-incubated with anti-LLO antibody or NRG for 1 h at 37 °C prior to incubation with RBC at 37 °C for 45 min. Lysis of RBC was determined at absorbance 541 nm (mean ± S.D., n = 3). Neutralization of hemolytic activity of LLO by the antibody was determined from a reduction of lysis of RBC. (**B**) RAW264.7 cells were cultivated on sterilized glass slides. The cells were treated with the antibody, NRG or PBS and infected simultaneously with DsRedEx-expressing *L. monocytogenes*. After incubation for 30 min, the extracellular bacteria were eliminated by gentamicin. At 4 h after infection, the cells were fixed and permeabilized. The cells were stained using mouse monoclonal anti-LAMP1 antibody and AlexaFluor 488-conjugated donkey anti-mouse IgG. DAPI was used to stain cell nucleus. Fluorescent signals were observed under fluorescence microscope. (**C**) Percentages of *L. monocytogenes* localized in lysosome and cytoplasm were quantified from at least 100 areas of 3-independent experiments (mean ± S.D.). *P < 0.01.

Prior to investigate neutralizing activity of anti-LLO antibody, recombinant LLO (rLLO) was prepared and hemolytic activity of rLLO was examined using sheep red blood cells (RBC). The hemolytic activity of LLO was concentration-dependent (see Supplementary Fig. S4). rLLO (1.0 μg/ml) was then pre-incubated with anti-LLO antibody and neutralization of rLLO activity was observed. As shown in Fig. 3A, lysis of RBC was reduced by pre-incubation of rLLO with anti-LLO antibody but not with NRG. The results demonstrate that anti-LLO antibody neutralizes LLO activity. We expected that anti-LLO antibody may enter into the host cells and suppress lysosomal escape of *L. monocytogenes* infection. Thus, co-localization of *L. monocytogenes* with lysosome in RAW264.7 cells was observed. As expected, bacterial number in the antibody-treated cells reduced comparing to phosphate-buffered saline (PBS)- or NRG-treated cells (Fig. 3B) and the percentage of *L. monocytogenes* localized in lysosome increased in the presence of anti-LLO antibody (Fig. 3B,C). Although the bacteria in anti-ActA antibody-treated cells also reduced, they were located in the cytosol. These results suggested that anti-LLO antibody neutralizes LLO activity and inhibits the bacterial escape from lysosomal vacuoles.

### Neutralizing activity of anti-ActA antibody

**Figure 4:**
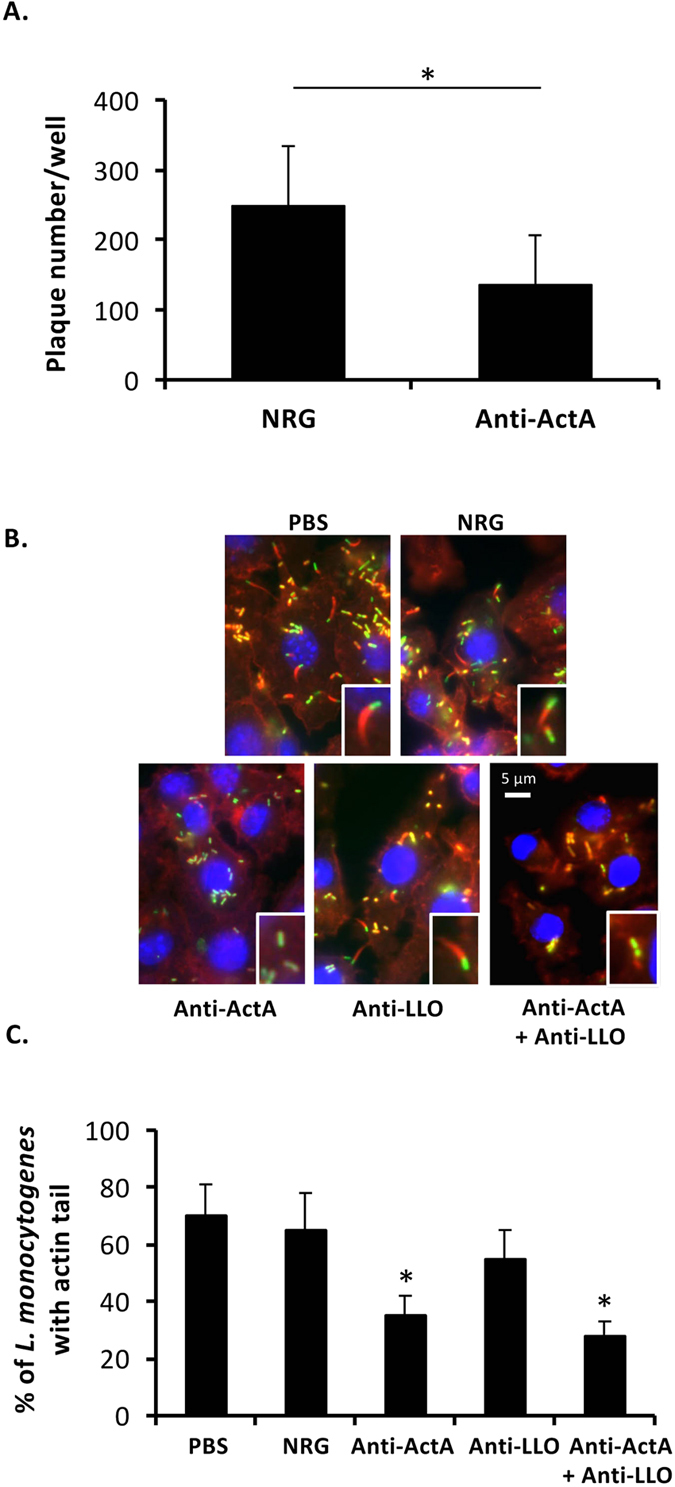
Neutralizing activity of the anti-ActA antibody. (**A**) NMuLi cells were treated with the antibody or NRG and infected with simultaneously with *L. monocytogenes* at MOI 10. Plaques under noble agar were enumerated on day 3 after infection. NRG was used as a control (mean ± S.D., n = 6). *P < 0.05. (**B**) RAW264.7 cells were cultivated on sterilized glass slides. The cells were treated with the antibody, NRG or PBS and infected simultaneously with YFP-expressing *L. monocytogenes*. After incubation for 30 min, the extracellular bacteria were eliminated by gentamicin. At 6 h after infection, actin tail was stained using rhodamine-conjugated phalloidin. DAPI was used to stain cell nucleus. (**C**) Percentage of *L. monocytogenes* containing actin tail was quantified from at least 100 areas of 3-independent experiments (mean ± S.D.). *P < 0.01.

Activity of ActA which promotes cell-to-cell spread was examined by plaque assay. On day 3 after infection into NMuLi cells, the number of plaque was significantly reduced by anti-ActA antibody comparing to NRG (Fig. 4A). Actin polymerization was then observed by immunostaining of listerial infected RAW264.7 cells. Defect in actin tail formation around listerial cells was found in the cell treated with anti-ActA antibody (Fig. 4B). Percentage of listerial cells localized with actin tail was significantly reduced by anti-ActA antibody (Fig. 4C). These results suggested that anti-ActA antibody neutralizes ActA activity, inhibits actin tail formation and attenuates cell-to-cell spread.

## Discussion

*L. monocytogenes* has long served as a model pathogen for elucidating many aspects between intracellular bacteria and host immune response^15^. Although *L. monocytogenes* is able to induce robust cytokines and T cell-mediated response^13^,^14^, it has evolved mechanisms to evade and modulate host immunity^15^. *L. monocytogenes* invades and replicates intracellularly resulting in an evasion and modulation of humoral immune response^3^. Due to its intracellular niche, B cells have low opportunity to encounter antigens. Antibody production during *L. monocytogenes* infection are very low and do not appear to play a significant role in the clearance of *L. monocytogenes*. Serum transfer from infected mice does not improve outcome of infected naïve mice^15^. Moreover, immunization of mice with neither heat-killed bacteria nor LLO-deficient mutant provide protective immunity^16^,^21^. It might due to these bacteria secreting or containing less secreted virulence factors. For these reasons, secreted virulence factors are expected to be appropriated antigens which provide protective antibodies against *L. monocytogenes*.

In the present study, we prepared antibodies against secreted virulence factors of *L. monocytogenes* in rabbits and passive immunization of these antibodies was performed. Antibodies against FbpA and p60 are expected to protect listerial adhesion to host cells, whereas antibodies against LLO and PI-PLC are candidates to block lysosomal escape. In addition, antibody against ActA is a candidate to prohibit cell-to-cell spread. Although anti-LLO antibody has been reported to provide protection^18^, not only LLO but also phospholipases are required to mediate lysosomal escape and intracellular growth^22^. Thus, the most efficient protective effect is expected from combination of antibodies against several virulence factors. Antibodies against FbpA, p60 and PI-PLC did not show protective effect in mouse model (see Supplementary Fig. S1A). To internalize into non-professional phagocytic cells, several adhesins and invasins play a crucial role. However, these factors are not considered to be required for internalization into professional phagocytes. In our experimental model, *L. monocytogenes* was infected intravenously. Thus, *L. monocytogenes* in blood circulation might be efficiently engulfed by macrophages without a significant mediation by FbpA and p60. In addition to LLO, PI-PLC contributes to escape from vacuoles^22^. However, another broad-range phospholipase C (PC-PLC) may be able to compensate the activity of PI-PLC. As part of its intracellular growth cycle, *L. monocytogenes* cells spread and are taken up directly by neighboring cells. During this process, double membrane vacuoles, called secondary vacuoles are generated^1^. To escape from these vacuoles, LLO is required to perforate the outer membrane^23^ whereas PI-PLC and PC-PLC individually act on the inner membrane^24^,^25^. Gründling and colleagues have demonstrated that in absence of PI-PLC, *L. monocytogenes* can escape from vacuoles mediating by PC-PLC^11^. To support this assumption, bacterial loads in the organs of mice administered with anti-FbpA, anti-p60 and anti-PI-PLC antibody is required in future.

Anti-ActA and anti-LLO antibodies could protect mice from listerial infection and the most efficiency was found from the combination of these antibodies (Fig. 1). This efficiency reduced when these antibodies were administered after infection for 6 h (see Supplementary Fig. S1B). This result suggested that administration of the antibodies as early as listerial infection is important. Furthermore, the effect of these antibodies was reduced in IFN-γ^−/−^ mice as well as in TNF-α^−/−^ mice. This result suggests that protective effect by passive immunization of anti-ActA and anti-LLO antibodies requires IFN-γ and also TNF-α^19^,^20^ (see Supplementary Fig. S2). However, the survival of IFN-γ^−/−^ mice and TNF-α^−/−^ mice remained 40% when immunization with anti-ActA and anti-LLO antibodies. These results suggest that the antibodies also act by a mechanism independent of IFN-γ and TNF-α.

Anti-ActA and anti-LLO antibodies were not required for adhesion (see Supplementary Fig. S3) and invasion (Fig. 2A). On the other hand, we clearly demonstrated that this protective effect is directly mediated by neutralization activities of ActA (Fig. 4) and LLO (Fig. 3). These antibodies could enter into the host cells either by endocytosis or phagocytosis with listerial cells (Fig. 2B,C). Our study suggests that anti-LLO antibody neutralizes LLO activity in lysosomal vacuoles, whereas anti-ActA antibody in the cytosol plays an important role to prohibit actin tail formation and cell-to-cell spread. Anti-LLO antibody alone could not provide a completely protective effect against *L. monocytogenes* infection. Some listerial cells were able to escape from lysosomes (Fig. 3B,C). Therefore, combination of anti-LLO antibody with anti-ActA antibody is a critical point. A study demonstrated that actin tail polymerization by ActA is required for evasion of autophagy^26^. Therefore, anti-ActA antibody may not only disturb actin polymerization, but also may promote localization of intracytosolic *L. monocytogenes* with autophagosomes. Clearance of intracellular *L. monocytogenes* mediated by these antibodies might be involved in autophagy because recent studies reported that autophagy is induced by IFN-γ and TNF-α stimulation and restricts intracellular growth of *L. monocytogenes*^27^,^28^. However, anti-ActA antibody alone also could not provide a completely protective effect, suggesting that only defect of actin tail is not enough for listerial clearance. In this situation, *L. monocytogenes* may be able to escape from autophagolysosomes by LLO. Therefore, anti-LLO and anti-ActA antibody may support each other to provide the most beneficial effect. For future achievement, LLO/ActA mutants and other listerial strains are required to address the specificity of antibodies.

Cell mediated immunity is an immune response that provides the protective effect against intracellular bacteria. However, our finding in the present study demonstrates that antibody approach is still useful for neutralizing the activities of virulence factors. Antibodies can be used for preventive and therapeutic applications against *L. monocytogenes* infection and this data may provide an idea for further applications to other intracellular bacteria.

## Materials and Methods

### Bacterial strains, plasmids, and growth conditions

*L. monocytogenes* 1b 1684 was grown in tryptic soy broth (BD Biosciences, Sparks, MD) at 37 °C and stored at −80 °C until use^29^. pJEBAN3 plasmid encoding yellow fluorescent protein (YFP) and pJEBAN6 plasmid encoding red fluorescence protein (DsRedEx), was transferred into *L. monocytogenes* by electroporation to give yellow and red fluorescent strain, respectively^30^. *L. monocytogenes* harboring pJEBAN3 or pJEBAN6 plasmid was cultured in tryptic soy broth supplemented with 5 μg/ml erythromycin (Wako Pure Chemical Industries, Osaka, Japan).

### Mice

C57BL/6 mice were purchased from Clea Japan Inc., Tokyo, Japan. IFN-γ^−/−^ and TNF-α^−/−^ mice (C57BL/6 background) were developed as previously reported^31^,^32^. Mice were maintained under specific-pathogen-free conditions in the Institute for Animal Experimentation, Hirosaki University Graduate School of Medicine. All animal experiments in this study were performed in accordance with the Guidelines for Animal Experimentation and were approved by the Animal Research Committee of Hirosaki University (approval number M08022).

### Cell lines and culture conditions

Mouse macrophage cell line RAW264.7 cells and mouse hepatocyte cell line NMuLi cells (Dainippon Sumitomo Pharma, Osaka, Japan) were cultured at 37 °C under 5% CO_2_ in Dulbecco’s modified Eagle medium (DMEM; Nissui Pharmaceutical Co., Tokyo, Japan), supplemented with 10% fetal bovine serum (FBS; JRH Biosciences, Lenexa, KS), and 0.03% of L-glutamine (Wako).

### Preparation of recombinant virulence factors of *L. monocytogenes*

Genomic DNA of *L. monocytogenes* 1b 1684 was used as template for amplification of virulence gene by PCR. Primers used in this study are shown in Table 1. Genes encoding FbpA, LLO and PI-PLC were cloned into pET15b plasmid (Merck Chemical KgaA, Darmstadt, Germany) and transferred to *Escherichia coli* Rosetta (DE3) pLysS. Genes encoding p60 and truncated ActA which lacks transmembrane domain at C-terminal were cloned into an pGEX-6p-1 plasmid (GE Healthcare Bio-Sciences, Tokyo, Japan) and transferred to *E. coli* DH5-α. *E. coli* Rosetta (DE3) pLysS harboring recombinant plasmid was grown in Luria-Bertani broth (LB; BD Biosciences) containing 100 μg/ml ampicillin and 34 μg/ml chloramphenicol. *E. coli* DH5-α harboring recombinant plasmid was grown in LB broth containing 100 μg/ml ampicillin. Production of recombinant protein was induced by addition of 1 mM isopropylthiogalactopyranoside (Wako). Histidine-tagged recombinant proteins from *E. coli* Rosetta (DE3) pLysS were purified using Talon 6× histidine affinity column (Takara Bio Inc., Ohtsu, Japan). To remove histidine tag, thrombin digestion was performed using thrombin cleavage capture kit (Novagen, Madison, WI). GST-fusion proteins from *E. coli* DH5-α were purified and GST was removed by using the bulk GST purification modules (Amersham Pharmacia Biotech) according to the manufacturer’s instructions. The purified proteins were analyzed by SDS-PAGE and quantified by Bradford protein assay (Bio-Rad, Richmond, CA). The final preparations were stored at −20 °C until use.

### Preparation of antibodies against virulence factors of *L. monocytogenes*

Purified recombinant virulence factors were used as antigens for preparing antibodies. Rabbits were immunized with 1 mg of each virulence factor plus 1 ml of Imject alum (Pierce Biotechnology Inc., Rockford, IL) three times at 2-week intervals. Serum was obtained 1 week after the final immunization. Immunoglobulin was concentrated by ammonium sulphate precipitation and purified using DEAE-Sephacel resin (GE Healthcare Bio-Sciences) according to the standard protocol. Briefly, ammonium sulphate was added to obtain 50% saturation. After being stirred for 30 min at 4 °C, the solution was centrifuged at 23,000 × *g* for 20 min. The pellet was then dissolved in PBS and the process of ammonium precipitation was repeated. Re-dissolved pellet was dialyzed against PBS and the proteins were applied to DEAE-Sephacel column pre-equilibrated with 20 mM Tris-HCl, pH 8.0. After washing, the proteins were eluted with 300 mM NaCl. The fractions containing immunoglobulin were confirmed by SDS-PAGE. The immunoglobulin was pooled, concentrated and the buffer was exchanged with PBS using Vivaspin filter units (Sartorius AG, Goettingen, Germany). Titer of antibodies was determined by enzyme-linked immunosorbent assay (ELISA). Protein was quantified by Bradford protein assay and the antibodies were stored at −80 °C until use.

### *L. monocytogenes* infection *in vivo*

Mice were administered with 1 mg of the antibody 24 h before *L. monocytogenes* infection. NRG was administered as a negative control. NRG was prepared from normal rabbit sera using the same protocol as that for the antibody. C57BL/6 mice were infected intravenously with 1 × 10^6^ CFU of *L. monocytogenes*, whereas IFN-γ^−/−^ and TNF-α^−/−^ mice were infected intravenously with 1 × 10^5^ CFU of *L. monocytogenes*. Survival of mice was observed and the number of viable bacteria in liver and spleen was determined as previously described^33^. For combination of antibodies, 0.5 mg of each antibody were mixed to obtain final amount of 1 mg.

### *L. monocytogenes* infection *in vitro*

RAW264.7 cells were seeded on 24-well culture plates (Asahi Glass, Tokyo, Japan) at 1 × 10^6^ cell/well in DMEM supplemented with 10% (v/v) FBS. Cells were treated with 100 μg of the antibody and infected simultaneously with *L. monocytogenes* at multiplicity of infection (MOI) 10. After incubation for 30 min, the extracellular bacteria were eliminated with 50 μg/ml gentamicin (Wako). The intracellular bacteria were enumerated by serial dilution and spread on tryptic soy agar (TSA) plates. For adhesion assay, RAW264.7 cells were seeded and incubated with the antibody and *L. monocytogenes* at MOI 10 as described above. NMuLi cells were seeded at 5 × 10^5^ cell/well and incubated with the antibody and *L. monocytogenes* at MOI 100. After incubation for 15 min, the non-adhesive bacterial cells were washed with PBS for 5 times. The adhesive bacterial cells were enumerated on TSA plates. For combination of antibodies, 50 μg of each antibody were mixed to obtain a final amount of 100 μg.

### Internalization of antibodies into RAW264.7 cells

RAW264.7 cells were cultivated on sterilized coverslips and treated with the antibody only or the antibody and DsRedEx-expressing *L. monocytogenes* at MOI 10. When indicated, the cells were treated with 10 μM cytochalasin D for 24 h before antibody treatment. After the antibody treatment, the cells were washed and fixed with 4% paraformaldehyde (Wako) for 15 min at room temperature and washed with PBS. The cells were then permeabilized with 0.5% Triton X-100 (Sigma-Aldrich Japan, Tokyo, Japan) in PBS. When indicated, permeabilization with Triton X-100 was omitted. After blocking with 2% donkey serum, antibodies were stained with AlexaFluor 488-conjugated donkey anti-rabbit IgG (Molecular Probes, Eugene, OR) according to the manufacturer’s instruction and counterstained with 4′,6-diamidino-2-phenylindole (DAPI; Dojindo Laboratories, Kumamoto, Japan). Fluorescent signals were observed under fluorescence microscope (BZ-X700; Keyence, Osaka, Japan).

### LLO-neutralizing activity of the anti-LLO antibodies

RBC from sheep (Nihon Biotest Research; Tokyo, Japan) were collected by centrifugation at 2,200 × *g*, 4 °C for 10 min, and diluted in PBS. To observe hemolytic activity of LLO, recombinant LLO was diluted in PBS and incubated with RBC at 37 °C for 45 min. Then, lysis of RBC was determined at absorbance 541 nm. To observe neutralizing activity by the antibody, recombinant LLO (1.0 μg/ml) was pre-incubated with anti-LLO antibody for 1 h at 37 °C. Neutralization of hemolytic activity of LLO by the antibody was determined from a reduction of lysis of RBC.

### Effect of antibodies to lysosomal escape

RAW264.7 cells were cultivated on sterilized coverslips. The cells were treated with the antibody and infected with DsRedEx-expressing *L. monocytogenes* as described above. After incubation for 30 min, the extracellular bacteria were eliminated by gentamicin. At 4 h after infection, the cells were fixed with 4% paraformaldehyde for 15 min at room temperature and washed with PBS. The cells were then permeabilized with 0.5% Triton X-100 in PBS and blocked with 2% donkey serum. Lysosomal vacuoles were stained with mouse monoclonal anti-LAMP1 antibody (Abcam, Cambridge, MA), and AlexaFluor 488-conjugated donkey anti-mouse IgG (Molecular Probes) according to the manufacturer’s instruction. Counterstaining of nuclei by DAPI was then performed. Fluorescent signals were observed under fluorescence microscope.

### Plaque assay

NMuLi cells were plated on 24-well culture plates at 5 × 10^5^ cell/well in DMEM supplemented with 10% (v/v) FBS. The cells were treated with 100 μg of the antibody and infected simultaneously with *L. monocytogenes* at MOI 10. After incubation for 30 min, the extracellular bacteria were eliminated with 50 μg/ml gentamicin. The noble agar (0.9%) prepared in DMEM containing 10% (v/v) FBS and 50 μg/ml gentamicin was overlaid. After 3 days, numbers of plaques were enumerated.

### Effect of antibodies to actin tail formation

RAW264.7 cells were cultivated on sterilized glass slides and infected with YFP-expressing *L. monocytogenes*. After incubation for 30 min, the extracellular bacteria were eliminated by gentamicin. At 6 h after infection, the cells were fixed with 4% paraformaldehyde for 15 min at room temperature and washed with PBS. The cells were then permeabilized with 0.5% Triton X-100 in PBS. Actin filaments were stained with 100 nM rhodamine-conjugated phalloidin (Cytoskeleton Inc., Denver, CO) and counterstained with DAPI. Fluorescent signals were observed under fluorescence microscope.

### Statistical analysis

Data were expressed as means ± standard deviations (S.D.), and Student’s *t* test (one-tail analysis) was used to determine the significance of the differences between control and experimental group. Differences with *P* values < 0.05 were considered statistically significant. Survival rates were analyzed by Kaplan-Meier survival estimate method. The *P*-values were calculated by the log-rank test. Three times of experiments were repeated. The sample sizes are indicated in the figure legend.

## Additional Information

**How to cite this article**: Asano, K. *et al*. Passive immunization with anti-ActA and anti-listeriolysin O antibodies protects against *Listeria monocytogenes* infection in mice. *Sci. Rep.* **6**, 39628; doi: 10.1038/srep39628 (2016).

**Publisher's note:** Springer Nature remains neutral with regard to jurisdictional claims in published maps and institutional affiliations.

## Supplementary Material

Supplementary Figures

## Figures and Tables

**Table 1:** Primers used in this study.

Gene name	Gene product		Primer sequence (5′-3′)
*fbpA*	FbpA	Forward	CCCATATGAGTGGTCGCATTATGAAGATCC
		Reverse	CCGGATCCTTAATTTTTTAACTCTAAAACGAGCT
*hly*	LLO	Forward	CCCATATGGATGCATCTGCATTCAATAAAG
		Reverse	CCGGATCCTTATTCGATTGGATTATCTACTT
*plcA*	PI-PLC	Forward	CCCATATGTTCCCATTAGGCGGAAAAGCATA
		Reverse	CCCTCGAGTTAGTTGAATTTATTGTTTTTTATGATG
*iap*	p60	Forward	CCCGAATTCATGAAAAAAAGCAACTATCGCGGC
		Reverse	CCCGTCGACTTATACGCGACCGAAGCCAAC
*actA*	ActA	Forward	CCCGAATTCGCGACAGATAGCGAAGATTC
		Reverse	CGGCACGAGCGAAGCATTTACCTCTTCAC

Underlines indicate restriction enzyme recognition sites.
