# Supplementary material for: Passive immunization with anti-ActA and anti-listeriolysin O antibodies protects against Listeria monocytogenes infection in mice
Source: Sci Rep. 2016 Dec 22;6:39628. doi: 10.1038/srep39628 (PMC5177876; doi:10.1038/srep39628)
Supplement: Supplementary Figures [file srep39628-s1.pdf]

Passive immunization with anti-ActA and anti-listeriolysin O antibodies protects against *Listeria monocytogenes* infection in mice

Krisana Asano[1], Hiroshi Sashinami[1], Arihiro Osanai[1], Shouhei Hirose[1], Hisaya K. Ono[2], Kouji Natira[3], Dong-Liang Hu[2], Akio Nakane[1,*]

Fig. S1 (Nakane)

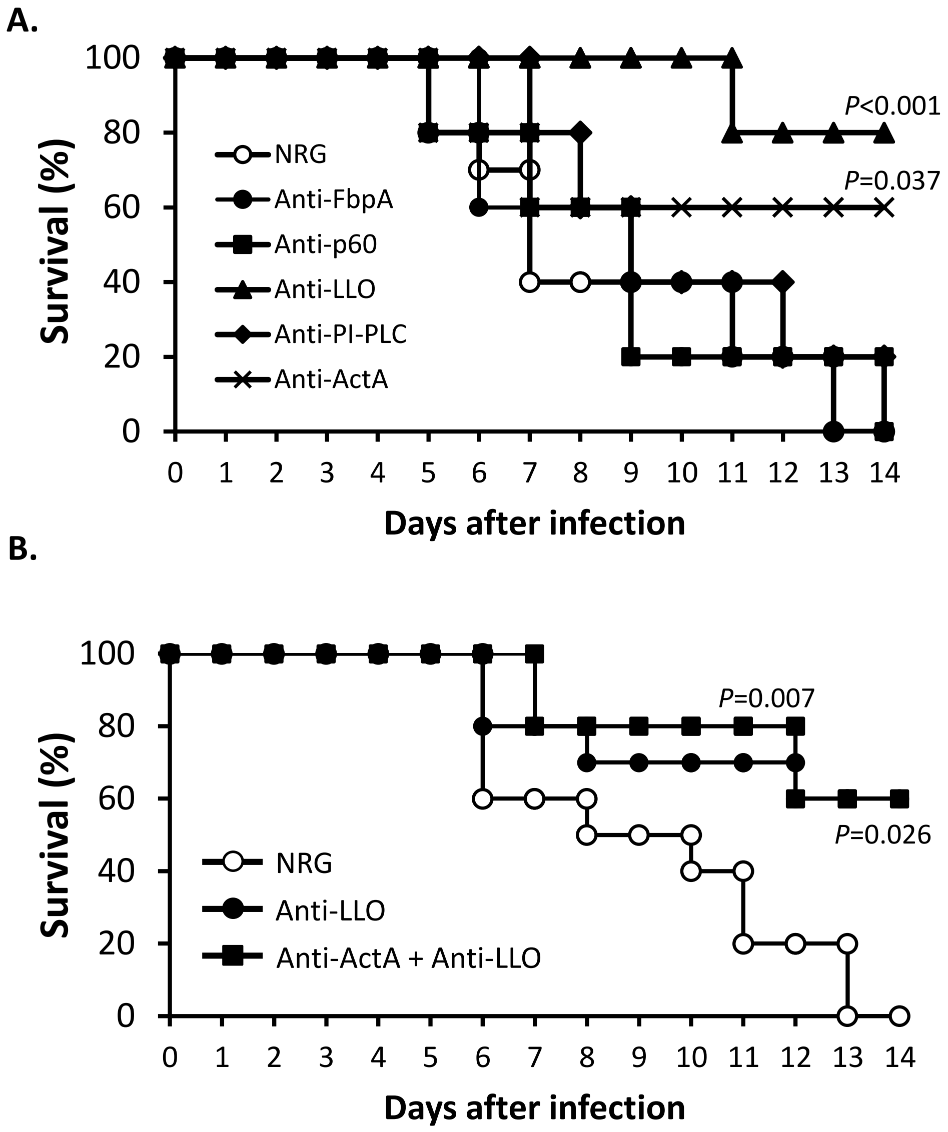

Figure S1. Effect of listerial antigen-specific antibodies on protection of mice against listerial infection. (A) Mice were administered intravenously with the antibody or NRG 1 mg/mouse. Mice were infected intravenously with 1 × 10^6 CFU *L. monocytogenes* 24 h later. (B) Mice were infected intravenously with 1 × 10^6 CFU *L. monocytogenes* for 6 h prior to intravenous administration with the antibody or NRG 1 mg/mouse. Survival was observed for 14 days (n=10). *P*-values calculated by log rank test are indicated.

Fig. S2 (Nakane)

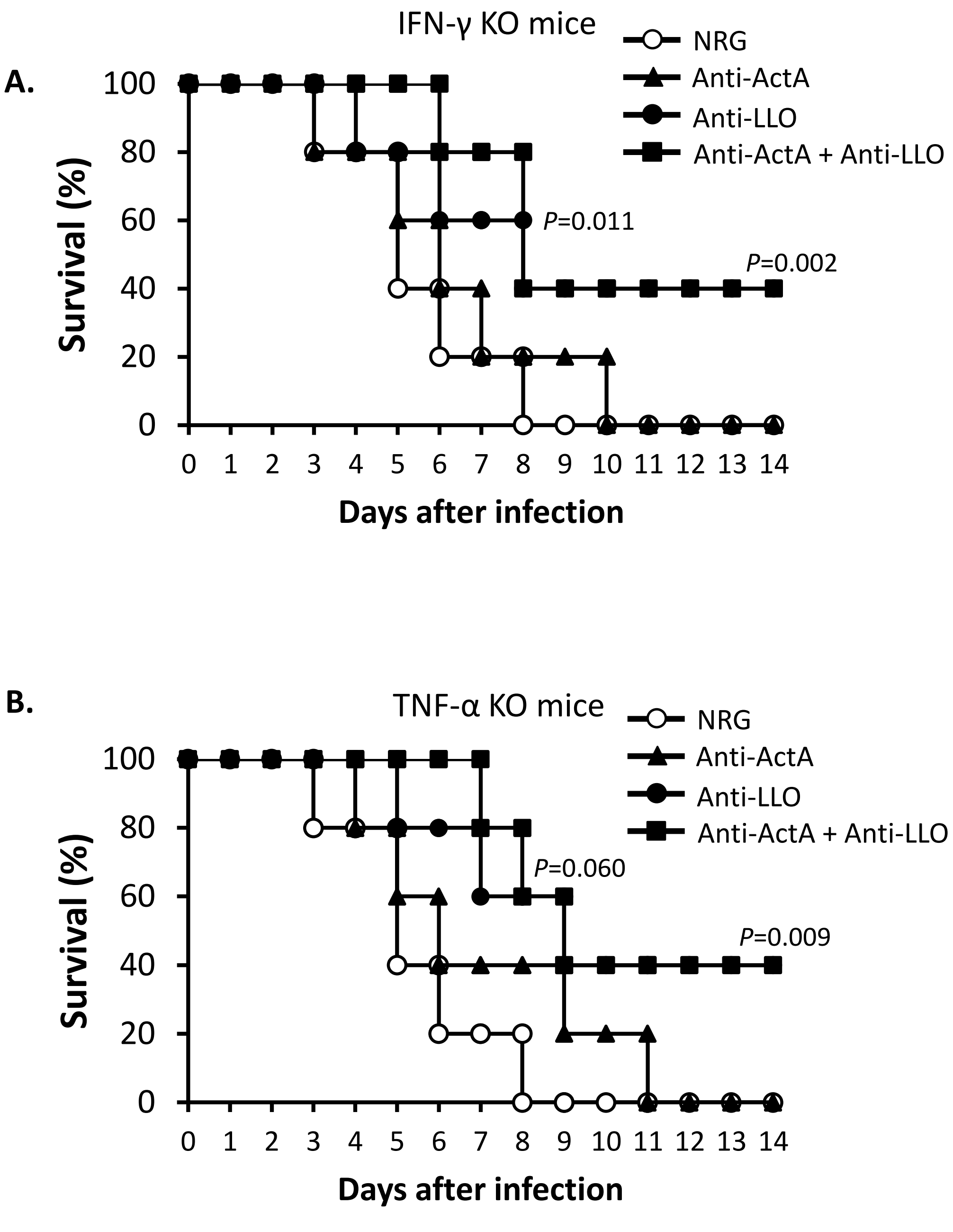

Figure S2. Passive immunization of anti-ActA and anti-LLO antibodies partially protects mice from listerial infection in IFN-$\gamma^{-/-}$ and TNF-$\alpha^{-/-}$ mice. (A) IFN-$\gamma^{-/-}$ and (B) TNF-$\alpha^{-/-}$ mice were administered intravenously with the antibody or NRG 1 mg/mouse. Mice were infected intravenously with 1×10^5 CFU *L. monocytogenes* 24 h later. Survival was observed for 14 days (n=10). *P*-values calculated by log rank test are indicated.

Fig. S3 (Nakane)

A.

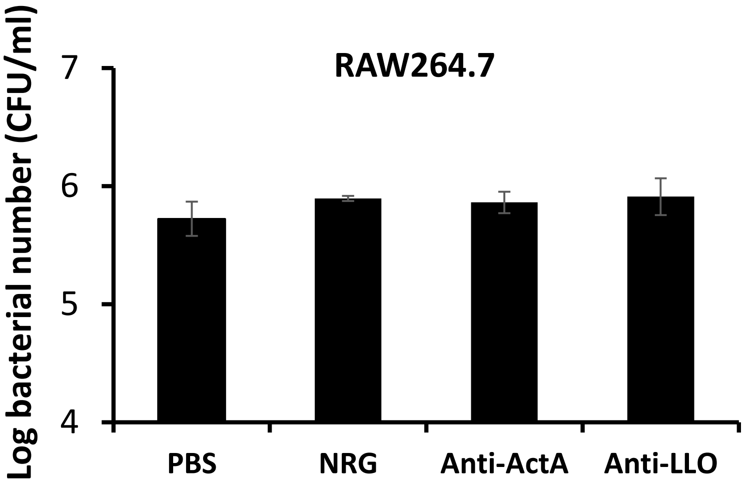

B.

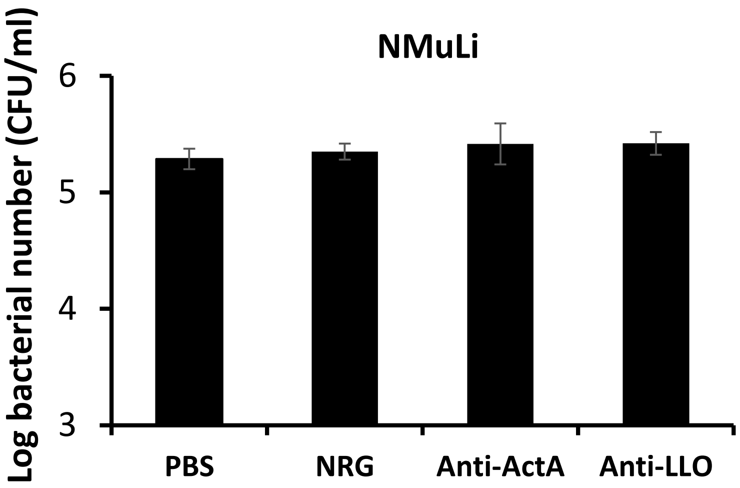

Figure S3. Antibodies did not promote bacterial adhesion into mouse macrophages and murine hepatocytes. (A) RAW264.7 and (B) NMuLi cells were plated on 24-well plates at 1×10^6 and 5×10^5 cell/well, respectively. The cells were treated with 100 µg/ml antibody, NRG or PBS and infected simultaneously with *L. monocytogenes*. RAW264.7 and NMuLi cells were infected with *L. monocytogenes* at MOI 10 and MOI 100, respectively. At 15 min of incubation, the non-adhesive bacterial cells were washed with PBS and the adhesive bacterial cells were enumerated on TSA plates (mean ± S.D., n=6).

Fig. S4 (Nakane)

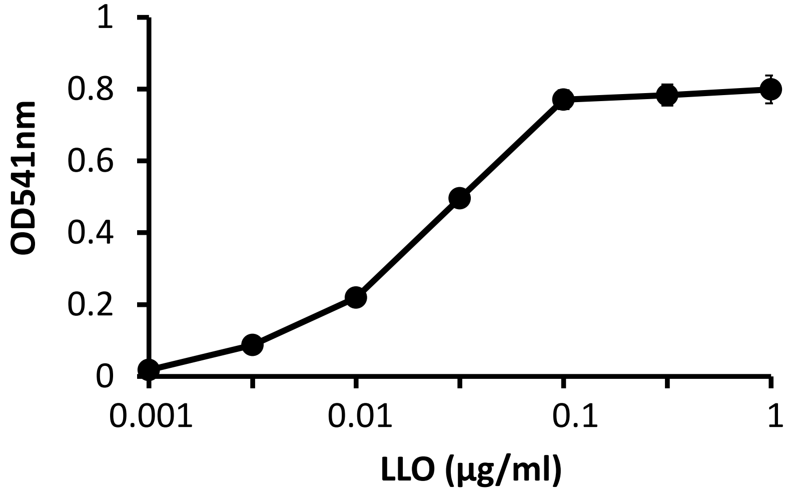

Figure S4. Hemolytic activity of rLLO. rLLO was diluted in PBS and incubated with sheep RBC at 37ºC for 45 min. Then, lysis of RBC was determined at absorbance 541 nm (mean ± S.D., n=6).
